# Endoscopic submucosal dissection of recurrent, circumferential, distal rectal tumor with severe submucosal fibrosis using multiple clip-line tractions

**DOI:** 10.1016/j.vgie.2023.08.003

**Published:** 2023-08-12

**Authors:** Darshan Parekh, Yohei Minato, Ken Ohata, Ryoju Negishi, Nao Takeuchi, Shunya Takayanagi, Marina Kim, Suryaprakash Bhandari

**Affiliations:** 1 Department of Endoscopy, NTT Medical Center Tokyo, Tokyo, Japan; 2 Department of Endoscopy, Thane Institute of Gastroenterology, Thane, Maharashtra, India; 3 Division of Gastroenterology & Hepatology, UMass Memorial Medical Center, Worcester, Massachusetts

## Abstract

Video 1 Endoscopic submucosal dissection for a recurrent, circumferential, distal rectal tumor.

Endoscopic submucosal dissection for a recurrent, circumferential, distal rectal tumor.

Endoscopic submucosal dissection (ESD) of large lesions is technically challenging. The difficulty increases when there is circumferential involvement, which has rarely been reported.^1^^,^^2^ Recurrent lesions are furthermore challenging because of severe submucosal fibrosis (SSF). We report successful en bloc removal of a recurrent, circumferential, rectal tumor using multiple clip-line tractions.

## Case

A 69-year-old woman was referred for management of a rectal lesion detected on surveillance colonoscopy.

**Figure 1:**
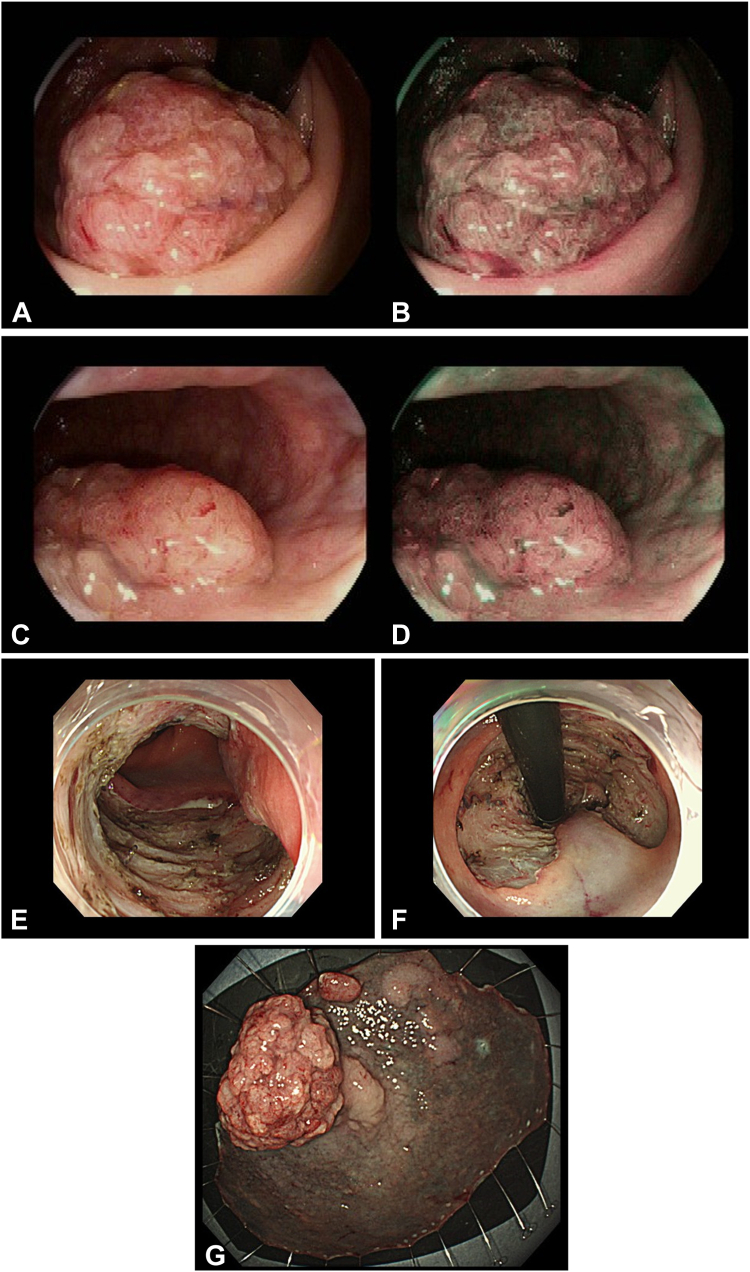
Past endoscopic submucosal dissection. **A,** Tumor in retroflexion view with white light. **B,** Tumor from retroflexion view with narrow-band imaging (NBI). **C,** Tumor from forward view with white light. **D,** Tumor from forward view with NBI. **E,** Ulcer from forward view. **F,** Ulcer in retroflexion. **G,** En bloc specimen.

She underwent ESD for a posterior wall rectal tumor 5 years prior (Fig. 1). The tumor measured 85 × 55 mm. After en bloc resection, 40 mg of triamcinolone was injected into the ulcer floor to prevent stricture formation. Pathology showed tubular adenoma with high-grade dysplasia and positive horizontal margins. She was undergoing yearly surveillance colonoscopies.

**Figure 2:**
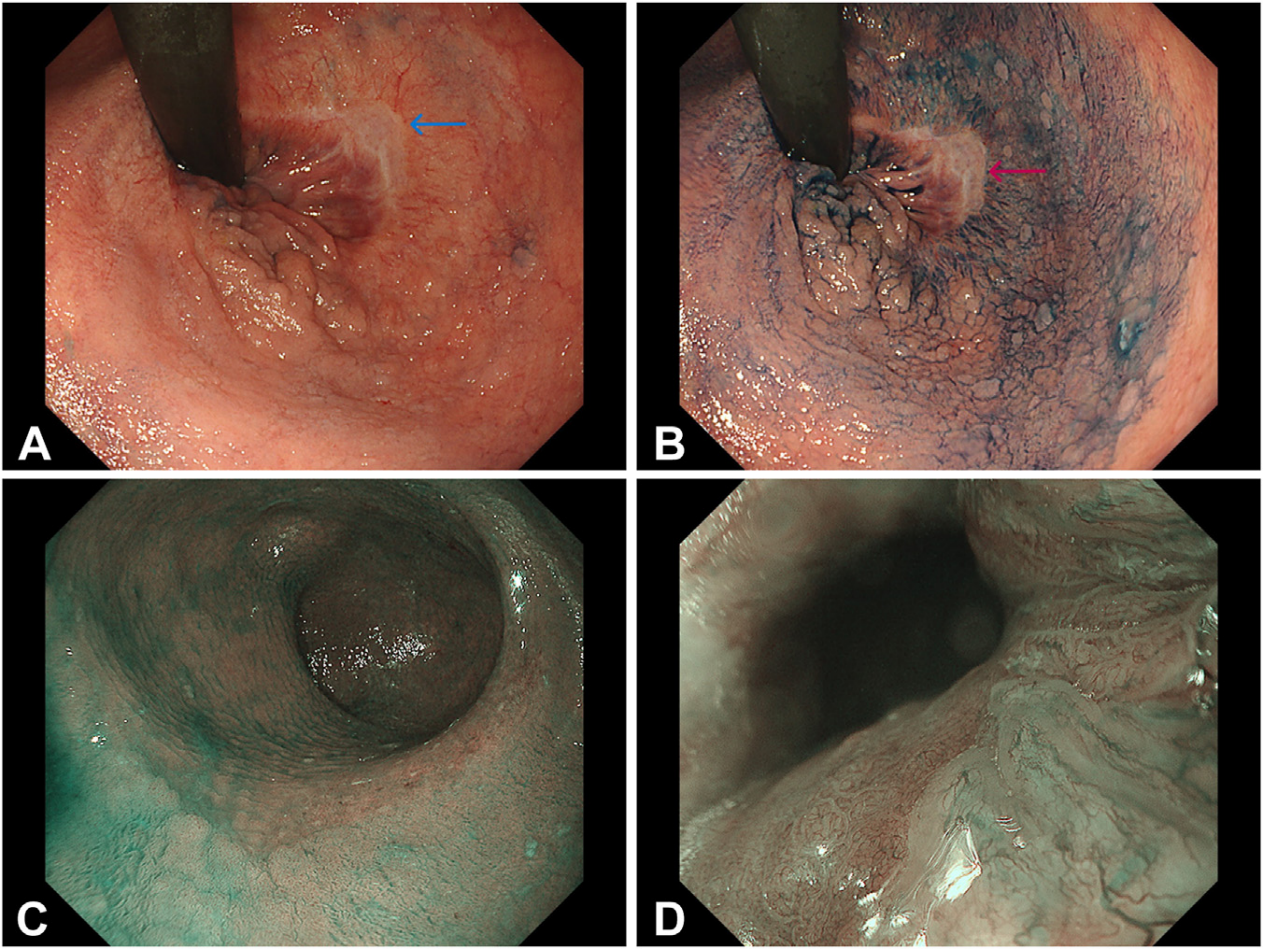
Circumferential, laterally spreading Japan Narrow-Band Imaging Expert Team type 2A tumor. **A,** Retroflexion view with white light (previous endoscopic submucosal dissection [ESD] scar indicated by *arrow*). **B,** Retroflexion view from chromoendoscopy with indigo carmine (previous ESD scar indicated by *arrow*). **C,** Narrow-band imaging (NBI) showing proximal demarcation line around 5 cm from the anal verge. **D,** NBI showing the distal demarcation line just above the anal verge.

Her latest colonoscopy revealed a circumferential, laterally spreading Japan Narrow-Band Imaging Expert Team type 2A tumor starting just above the anal verge and extending 5 cm proximally with the previous ESD’s scar (Fig. 2). ESD was performed because the patient preferred endoscopic management (Video 1, available online at www.videogie.org).

## Procedure

**Figure 3:**
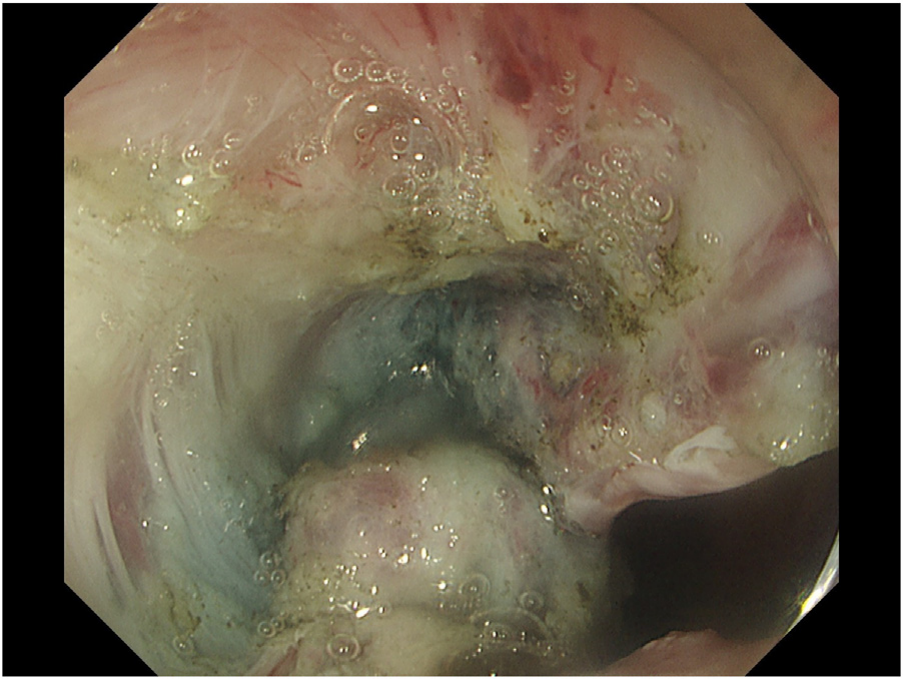
Severe submucosal fibrosis from previous endoscopic submucosal dissection scar.

**Figure 4:**
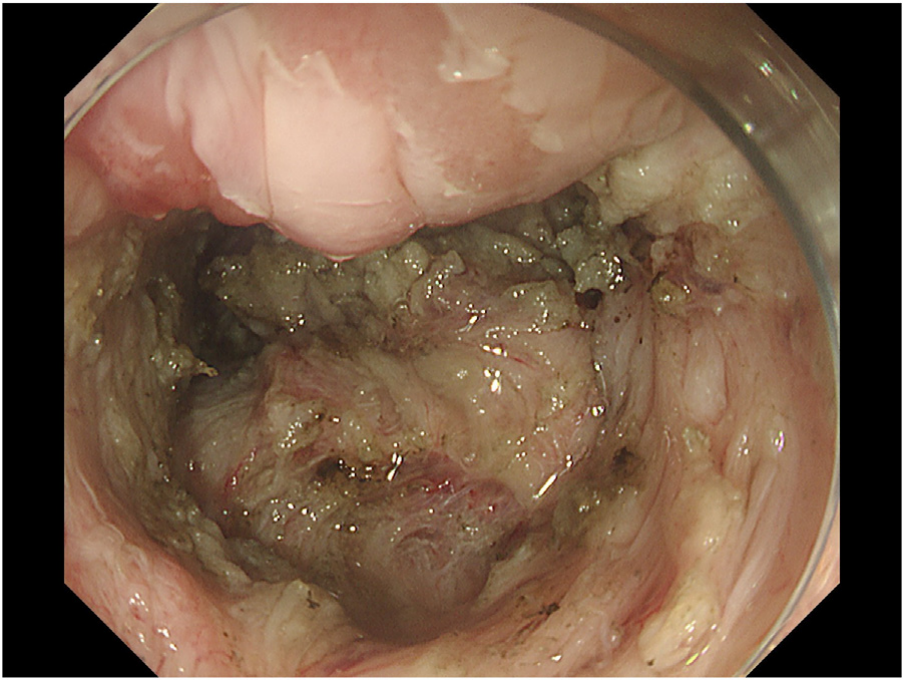
Lesion falling into lumen because of cutting of anchoring mucosa and submucosa.

**Figure 5:**
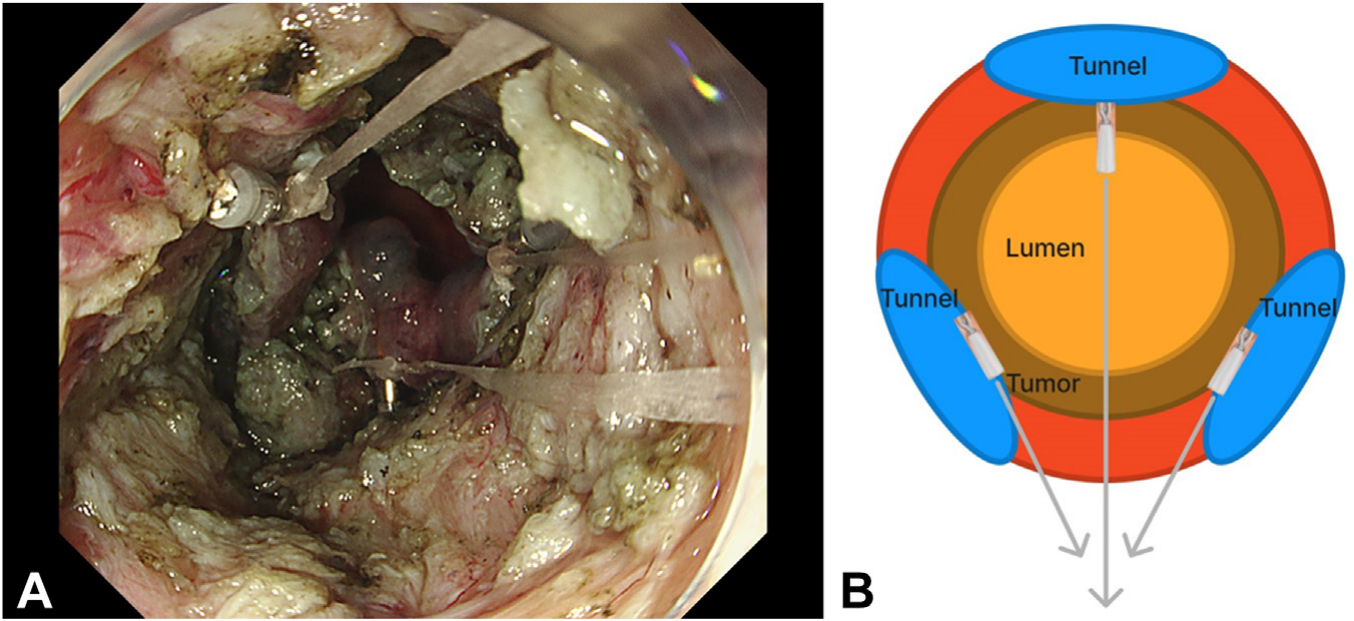
**A,** Three clip-line tractions applied in a triangular manner. **B,** Schematic showing multiple tunnels and clip-line tractions.

ESD was planned using the multiple tunnel technique.^2^ However, since the lesion was just above the anal verge, and because of the SSF from the previous ESD and steroid injection (Fig. 3), neither mucosa nor adequate submucosa could be preserved. This led to the lesion falling proximally into the lumen, impairing submucosal visibility (Fig. 4). Thus, 3 clip-line tractions^3^ were placed in a triangular manner over the lesion at the beginning of each tunnel (Fig. 5). En bloc resection was achieved, and tractions were removed from the specimen. Again, 40 mg of triamcinolone was injected equally into all 4 quadrants of the ulcer floor to prevent stricture formation. The procedure time was 300 minutes. No antibiotics were administered during or after the procedure.

## Outcome

**Figure 6:**
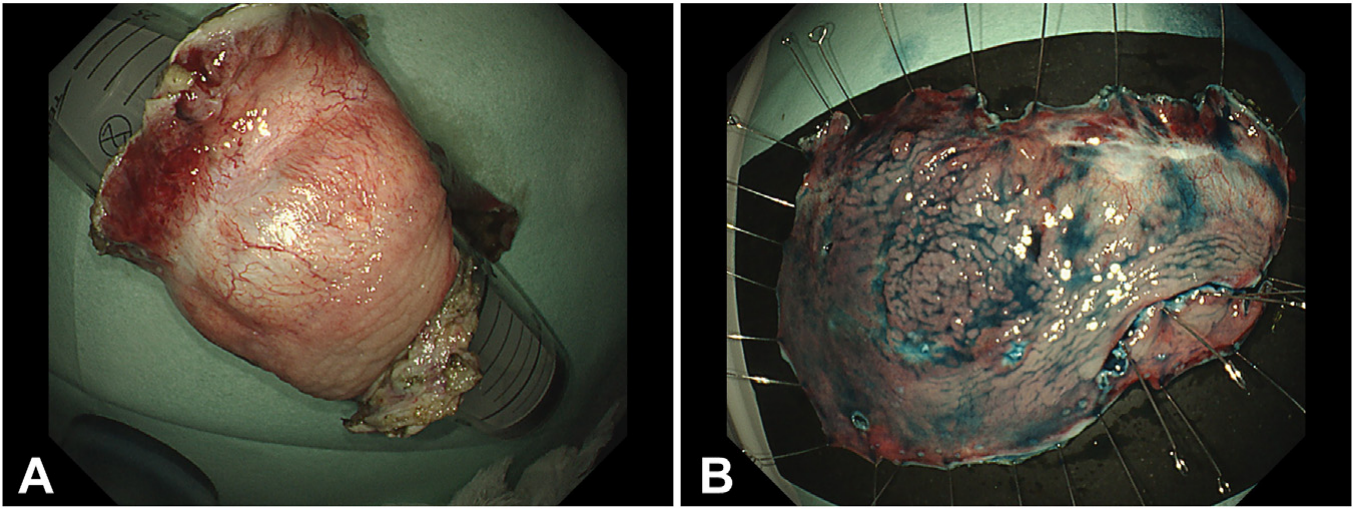
Specimen. **A,** Circumferential en bloc resection. **B,** Cut open (110 × 75 mm).

**Figure 7:**
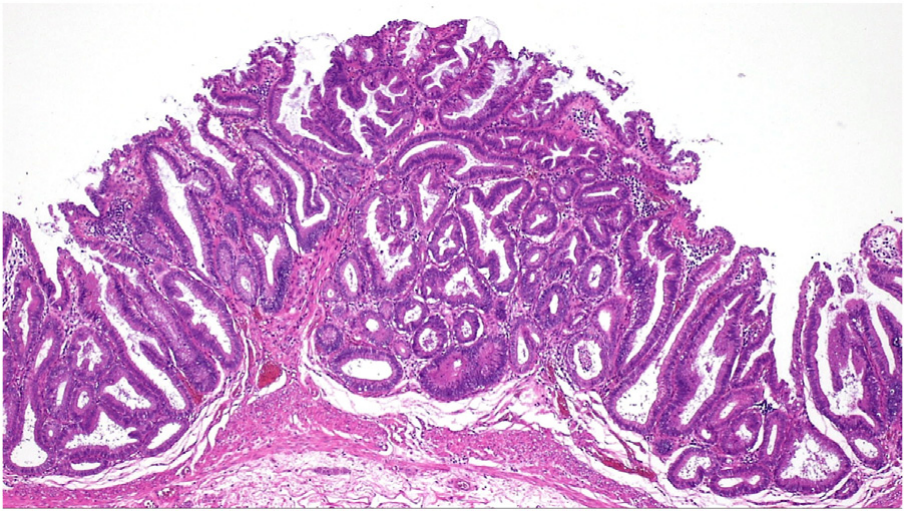
Histopathology. Tubular adenoma with high-grade dysplasia (all margins negative) (H&E, orig. mag. ×40).

**Figure 8:**
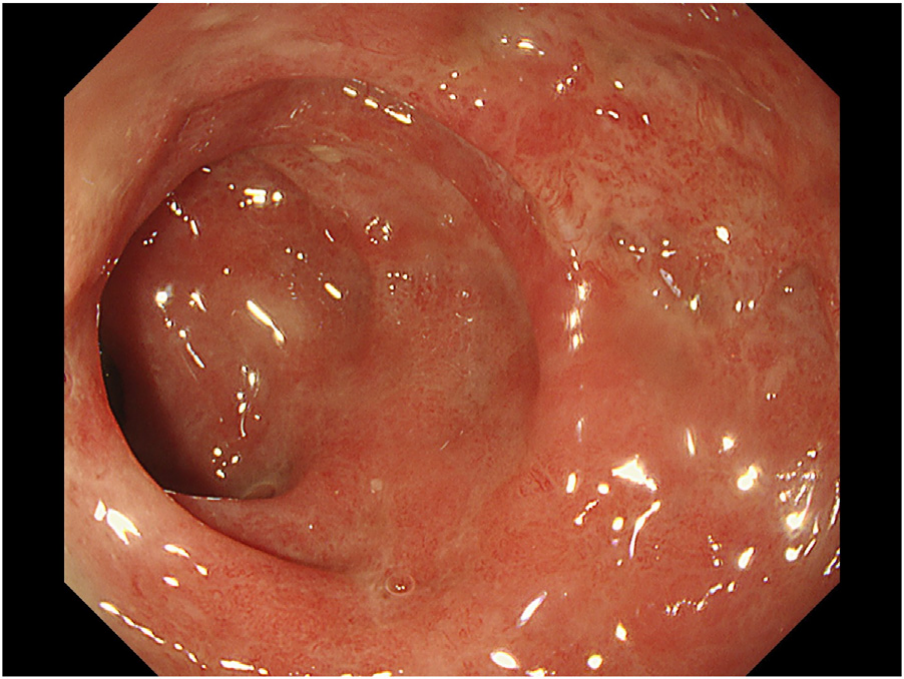
Follow-up colonoscopy after 45 days. No stricture, complete healing, no residual tumor.

The specimen measured 110 × 75 mm (Fig. 6). There were no adverse events during and immediately after the procedure. The patient was discharged on day 5. Pathology reported a tubular adenoma with high-grade dysplasia with negative margins (Fig. 7). A follow-up endoscopy after 45 days revealed no stricture with complete mucosal healing and no residual lesion (Fig. 8).

## Discussion and Conclusion

The GI lumen has a limited size, making resection of a circumferential lesion difficult. To prevent the lesion falling into the lumen by gravity and obscuring the view, techniques such as the multiple tunneling technique,^2^ pocket creation method,^4^ and palisade technique^5^ have been described.

The additional challenge in this case is SSF from the previous ESD and subsequent triamcinolone injection. The double tunnel method^6^ had been previously reported to manage SSF.

We decided to use the multiple tunnel technique. Intraprocedure, it was not possible to preserve mucosa because of the distal extent of the lesion located just above the anal verge leading to a very narrow space. We modified our strategy to use the palisade technique. However, it was not possible to preserve adequate submucosal tissue because of the SSF and the angle of dissection at such a distal location being steeply downward. Therefore, the lesion collapsed into the lumen. Various reports have been published about traction techniques for ESD.^7^ We strategized to use multiple clip-line tractions^4^ to draw the lesion outward and created space as required by adjusting the direction of external pull. It facilitated visibility and managed the SSF.

Recent reports have described novel methods of using a cell sheet^8^ or a polyglycolic acid sheet with fibrin^9^ glue for post-ESD stricture prevention in the esophagus. There is no standardization regarding management of strictures after colorectal ESD. We used a triamcinolone injection as per protocol.^10^

In conclusion, recurrent, circumferential lesions are rare and very challenging. Such large rectal tumors can be removed en bloc safely by applying adequate traction at multiple points, even when located distally. It is important to have the knowledge and experience to apply different ESD techniques to manage such complex cases.

## Disclosure

The authors did not disclose any financial relationships.

## Supplementary data

Video 1 Endoscopic submucosal dissection for a recurrent, circumferential, distal rectal tumor.
